# Socioeconomic Status and Racial or Ethnic Differences in Participation: Web-Based Survey

**DOI:** 10.2196/11865

**Published:** 2019-04-10

**Authors:** Myoungock Jang, Allison Vorderstrasse

**Affiliations:** 1 University of Wisconsin-Madison Madison, WI United States; 2 Rory Meyers College of Nursing New York University New York City, NY United States

**Keywords:** survey, technology, race, socioeconomic status

## Abstract

**Background:**

Web-based survey data collection has been widely used because of its advantages, although attaining and retaining participants can be challenging. There are several factors associated with successful Web-based survey participation; yet little is known regarding racial or ethnic and socioeconomic differences in the progress of a Web-based survey.

**Objective:**

This study aimed to examine racial or ethnic and socioeconomic status (SES) differences in participation in a Web-based survey.

**Methods:**

We conducted a secondary data analysis of a study dataset containing information on parents of preschool children. We used 2 phases of Web-based surveys: (1) screening questions including race or ethnicity information and (2) full survey with a consent form. Once potential participants submitted the screening questions, including their racial or ethnic information, the team sent the full survey link to potential participants who met study eligibility criteria. We calculated the proportion of racial or ethnic groups in each of the following areas: consent, partial survey completion, and total survey completion.

**Results:**

A total of 487 participants (236 non-Hispanic white, 44 Hispanic, 137 black, and 70 Asian) completed initial screening questions, and a total of 458 participants met study eligibility criteria. Compared with black participants, non-Hispanic white and Asian participants were more likely to consent to participate in the study (odds ratio [OR] 1.73, 95% CI 1.08-2.78, *P*=.02; OR 2.07, 95% CI 1.04-4.13, *P*=.04, respectively). There was no racial or ethnic difference with respect to the completion of demographic questions or completion of a partial survey. Finally, compared with black participants, non-Hispanic white participants were more likely to complete the entire survey (OR 3.36, 95% CI 1.51-7.06, *P*<.001). With respect to SES, less educated non-Hispanic white participants were less likely to complete the survey compared with their counterparts with more education (OR 0.15, 95% CI 0.50-1.48, *P*<.001).

**Conclusions:**

We found a significant difference among racial or ethnic groups as well as different education levels in Web-based survey participation. Survey researchers need to consider the SES and racial or ethnic differences in Web-based survey participation and develop strategies to address this bias in participation and completion in their research.

## Introduction

Self-report surveys are often a main data collection or measurement strategy in quantitative research. The self-report data collection method is used not only for major nationally representative datasets such as National Health and Nutrition Examination Survey and the US Census [1,2] but also by researchers conducting individual research [3]. Due to numerous advantages such as convenience and cost-effectiveness, self-report surveys are frequently used to collect data from individuals [4].

There are challenges to promoting reliable and credible data collection by a self-report survey. A primary challenge for researchers employing self-report surveys is to encourage the target population to initiate and complete the survey as the method heavily relies on self-selection. Self-selection bias refers to when survey participants are allowed to decide whether or not they want to participate in a survey [5] and is often mentioned as a limitation on the generalizability of the results of survey studies [6]. Random sampling from the general population for survey research is a key method for enhancing the generalizability of the study outcomes. However, the convenience sampling method instead of random sampling is widely used despite the known weaknesses because of the ease of researchers’ access to the target population [7]. Even after researchers have accessed the target population, there are some challenges for both researchers and participants in completing the survey, which could influence the data collection procedures. The issues of a low completion rate and a low return rate even for nationally representative epidemiological studies are well documented in survey research [8]. For instance, the traditional method of administering surveys using paper and pencil could make the processes of data collection time-consuming, including survey preparation, survey distribution, survey turnaround or return, data entry, and data preparation [9].

More recently, Web-based survey methods with advantages over traditional paper surveys have been widely used. The identified advantages compared with the traditional paper survey method include more flexible design options, lower delivery cost, and less data entry time [10]. However, there are also several challenges that researchers often face when implementing a Web-based survey method for data collection. Similar to traditional survey methods, initiating and completing the survey can be still challenging. Participants who respond to survey research are often more motivated, more technologically savvy, and more actively engaged in their health as well as the topic being studied, which could result in self-selection bias [11]. There have been mixed reports in the literature regarding the differences in response rate between a Web-based survey and a traditional paper survey. Zuidgeest et al reported comparable response rates for a mixed-mode survey, which included a Web-based survey and a mailed questionnaire [12]. However, Aitken et al obtained a return rate from a Web-based survey that was much lower than the equivalent paper-based survey [13]. Thus, Web-based survey methods might be in a transitional phase as an alternative method to collect data; as such, their use, which can be conducted with several advantages such as a lower cost, might yield varying response rates [14].

For further advancement of Web-based survey, researchers need to understand that there are many factors that influence Web-based survey participation. First, a principal factor influencing an initiation of the Web-based survey includes survey target population characteristics such as age, gender, socioeconomic status (SES), and race or ethnicity. These characteristics might influence the potential respondent’s accessibility to the internet as well as their motivation to participate in a Web-based survey. Age might influence the response to a Web-based survey because of different rates of internet usage by age group; for example, according to the Pew Research Center, about 97% to 98% of adults aged 18-49 years use the internet, whereas only about 66% of adults aged 65 years and above use the internet [15]. Thus, younger potential participants might more frequently participate in a Web-based survey and be more familiar with the features of a Web-based survey method. Although gender, family income, and race or ethnicity are not known to be directly related to internet usage, those who have not completed high school are less likely to access the internet than college graduates (65% vs 97%) [15]. McDonald et al [16] targeted young college students to investigate tobacco use and assessed the response rate during the second phase of the data collection. They found that individuals who were male, black, or seeking a bachelor’s degree were less likely to complete the survey compared with those who were female, white, or seeking an advanced degree.

The methods by which a survey is presented and delivered as well as its features comprise another principal factor influencing completion of the survey. The presentation of a Web survey might be more flexible than a traditional paper version survey, including the proper implementation of skip patterns or branching items that could filter responses to questions within or subsequent to the trigger question [10]. Web-based survey instruments also include features such as a progress bar to help participants to track their progress in the survey, which might improve the likelihood that they will complete the survey by reducing the perception of task burden [17]. In terms of survey delivery, Sauermann et al [18] found that personalized survey contact (eg, using the first name of the participant when sending a reminder email), a lottery incentive for survey completion, and changing contact wording (ie, change the wording of each contact message to maintain respondent attention) were positively associated with response rate. Thus, factors related to the transition of a survey from a paper format to a Web-based version with more flexible approaches might influence the completion rate [10].

It has been emphasized to include socioeconomically disadvantaged populations and under-represented racial or ethnic minorities to health research to diminish health disparities and improve health equity. As internet and other technologies are ubiquitous in today’s society, researchers might anticipate that such technologies can diminish SES and racial or ethnic participation differences in participation in a Web-based survey. However, with the exception of specific studies of response rates, such as that of McDonald et al [16], there is limited information regarding SES and racial or ethnic differences in Web-based survey participation. Due to a paucity of information, it is important to understand whether the progress of participation in Web-based surveys is similar across socioeconomic and racial or ethnic groups.

This study aimed to examine the SES and racial or ethnic differences of participation progress in a publicly available Web-based survey.

## Methods

### Study Setting and Recruitment

We conducted a secondary analysis of data from a cross-sectional study that enrolled racially or ethnically diverse parents of preschool children to examine the relationship between parental psychological distress and parental feeding practices in families of preschool children and to understand parents’ practices in child feeding and food preparation [19]. Inclusion criteria were as follows: a participant was a parent or guardian of a child aged 2-5 years (1) who spoke and read English and (2) whose child did not have any chronic disease diagnosed by a health care provider that could affect his or her diet or body mass index.

We started our recruitment for the parent study at local community settings (including preschools, churches, and libraries) as well as community activity facilities (eg, Young Men’s Christian Associations) in the southeast area of the United States using convenience sampling methods. With permission from school authorities, several local preschool administrators and teachers informed families about the study using the study flyer (eg, sending the flyer home with each child). A research staff member visited the preschool as needed to introduce the study to potential participants when they dropped off or picked up their children. We identified ethnicity-specific churches (eg, Korean churches, black churches, and Hispanic churches) and asked them to post study flyers on their church bulletin boards. The research staff also visited ethnic grocery stores (ie, Hispanic and Asian stores) to post flyers with the permission of the owners. We asked each enrolled participant to mention our study to their friends, relatives, or other potentially eligible families. We also targeted local pediatric clinics to post the flyer in the waiting room. The research staff visited the clinics as needed to introduce the study and to give a flyer to potential participants when they visited their health care providers.

In addition, we posted the study flyer in online communities or other social media such as Facebook to enhance our reach to potential participants. To accelerate the recruitment, we also posted the flyer on Craigslist, which is a nationwide advertisement website for community residents. We selected at least one city from each state (excluding Hawaii and Alaska) and targeted major metropolitan cities to have a more socioeconomically and racially or ethnically diverse sample.

### Data Collection

Our initial goal was to recruit comparable participants across racial or ethnic groups to compare subgroup differences in the relationship between parental psychological distress and parental practices in feeding. Thus, we used a 2-phase Web-based survey developed in Research Electronic Data Capture (REDCap) hosted at Duke University [20], which included (1) screening questions, including race or ethnicity information, and (2) the survey, with a consent form preceding the survey. This strategy allowed us to identify the race or ethnicity of potential study participants before the initiation of the survey. Once potential participants accessed and completed the screening questions (using a publicly available survey link), including their race or ethnicity, we sent the full survey link to eligible participants within 12 hours of confirming their eligibility.

Once they consented, they could freely access the Web survey for up to a month to encourage their completion of the survey. We sent each participant at least one reminder email if they did not finish the survey within a week, using the individual’s first name and different wording for each reminder email. We sent up to 2 weekly reminder emails. The survey required approximately 30-40 min to complete. In brief, the main survey consisted of demographic questions including SES indicators (ie, annual family income and level of education) and other validated questionnaires to assess perceived stress (Perceived Stress Scale, 10 items) [21], parenting stress (Parental Stress Scale, 18 items) [22], sleep quality (Pittsburgh Sleep Quality Index, 19 items) [23], perceived depression (The Center for Epidemiological Studies Depression Scale, 20 items) [24], social support (Social Support Questionnaire-Shortened version, 12 items) [25], home food availability (Home Food Inventory, 13 major food categories) [26], feeding practices (subscales from Child Feeding Questionnaire, 12 items) [27], and child eating behaviors (Harvard Service Food Frequency Questionnaire) [28]. We provided a thank-you gift by mail (eg, water bottle or divided plate for children) to each participant who completed the survey.

### Data Analysis

We exported all data from REDCap and conducted data analysis using SPSS (version 24, IBM). First, we conducted descriptive data analyses of sample distributions and characteristics (ie, race or ethnicity, age, and gender) of those who at least completed the demographic questions, which were on the initial page of the main survey after they consented. We categorized the participants based on their eligibility, consent response, whether they completed demographic questions (initial section of the main survey), whether they completed at least half of the survey (partial survey), and whether they completed the entire survey. We used annual family income and the education level as proxy indices of SES. We calculated the proportions for each group by participants’ demographic characteristics (mainly SES and race or ethnicity). We then used logistic regression to test for any significant differences in terms of completing the survey across racial or ethnic and education groups. On the basis of different progress and completion rates, we treated black participants (for race or ethnicity) and those participants who had completed graduate school (for education level) as reference groups for the regression model. We then conducted a 3-factor Chi-square (*χ*^2^) analysis to explore survey participation by SES within each racial or ethnic group.

## Results

### Sample Characteristics

A total of 459 participants (223 non-Hispanic white [NHW], 42 Hispanic, 132 black, and 62 Asian) completed screening questions identifying their race or ethnicity and met study eligibility criteria (Table 1). Of these, a total of 310 participants consented to participate in the study, and 259 participants completed the demographic questions. Table 1 shows the demographic characteristics of those who consented and completed demographic information. Of those who completed demographic questions (n=259), 84.4% (221/259) were female; we had asked the primary caregiver, who tended to be the child’s mother, to participate in the survey. A majority of participants (81.3% [213/259]) were at least college graduates, and 33.4% (87/259) of the participants had an annual family income of US $80,000 or more.

### Proportion of Participation Among Different Racial or Ethnic Groups and Socioeconomic Status Groups

Table 2 shows the patterns of study participation by race or ethnicity and education.

There was a racial or ethnic difference in obtaining informed consent for participation. Among those who completed screening questions and were eligible, NHWs were 1.7 times more likely to consent to participate in the study than blacks (odds ratio [OR] 1.73, 95% CI 1.08-2.78, *P*=.02) and Asians were twice as likely to consent to participate in the study as black participants (OR 2.07, 95% CI 1.04-4.13, *P*=.04). However, there was no racial or ethnic difference in the rates of completion of both the demographic questions and of partial surveys. Finally, there was a significant difference among racial or ethnic groups for total survey completion rate. Compared with black participants, NHW participants were more likely to complete the entire survey (OR 3.26, 95% CI 1.51-7.06, *P*<.001).

**Table 1:** Sample characteristics (N=259).

Characteristics		n (%)^a^
**Sex**		
	Male	38 (15.6)
	Female	221 (84.4)
**Age group (years)**		
	≤30	61 (23.6)
	30-40	148 (57.1)
	40-50	47 (18.1)
	≥50	3 (1.2)
**Race or ethnicity**		
	Non-Hispanic white	134 (53.4)
	Hispanic or Latino	22 (8.8)
	Black	48 (19.1)
	Asian	35 (13.9)
**Education**		
	Less than or equal to high school graduate	48 (18.4)
	College graduate	143 (54.8)
	Graduate school graduate	70 (26.8)
**Annual family income**		
	≤US $19,999	25 (9.6)
	US $20,000-US $39,999	39 (15.0)
	US $40,000-US $59,999	53 (20.4)
	US $60,000-US $79,999	48 (18.5)
	US $80,000-US $99,999	31 (11.9)
	≥US $100,000	56 (21.5)

^a^Total numbers might vary because of missing values.

**Table 2:** Logistic regression for the relationships of race or ethnicity and education with Web-based survey participation.

Predictor			Beta	SE	Wald chi-square (*df*)	*P* value	OR^a^ (95% CI)
**Consent signed to participate**							
	**Race or ethnicity (black as a reference group)**						
		NHW^b^	0.55	0.24	5.17 (1)	.02	1.73 (1.08-2.78)
		Hispanic	0.24	0.38	0.42 (1)	.52	1.28 (0.61-2.69)
		Asian	0.73	0.35	4.32 (1)	.04	2.07 (1.04-4.13)
**Demographic data completion**							
	**Race or ethnicity (black as a reference group)**						
		NHW	0.42	0.35	1.44 (1)	.23	1.52 (0.77-3.01)
		Hispanic	−0.01	0.51	0 (1)	.98	0.99 (0.36-2.69)
		Asian	−0.03	0.44	0.01 (1)	.94	0.97 (0.41-2.30)
**Half of the survey completion**							
	**Race or ethnicity (black as a reference group)**						
		NHW	0.69	0.44	2.44 (1)	.12	1.99 (0.84-4.69)
		Hispanic	0.58	0.72	0.64 (1)	.42	1.78 (0.44-7.26)
		Asian	0.46	0.72	0.41 (1)	.52	1.58 (0.38-6.44)
	**Education (graduate school graduate as a reference group)**						
		High school graduate or less	−2.53	0.81	9.86 (1)	.001	0.08 (0.02-0.39)
		College education	−1.51	0.77	3.86 (1)	.05	0.22 (0.49-1.00)
**Total survey completion**							
	**Race or ethnicity (black as a reference group)**						
		NHW	1.18	0.39	9.02 (1)	<.001	3.26 (1.51-7.06)
		Hispanic	0.91	0.64	2.04 (1)	.15	2.48 (0.71-8.67)
		Asian	1.24	0.69	3.24 (1)	.01	3.44 (0.90-13.20)
	**Education (graduate school graduate as a reference group)**						
		High school graduate or less	−1.87	0.58	10.55 (1)	<.001	0.15 (0.50-0.48)
		College education	−0.68	0.53	1.63 (1)	.2	0.51 (0.18-1.44)

^a^OR: odds ratio. Models adjusted for age for both race or ethnicity and education of study participants.

^b^NHW: non-Hispanic white.

**Table 3:** Web-based survey participation by education level within each racial or ethnic group (N=259).

Categories and subcategories		Demographics completion, n (%)	Partial survey completion, n (%)	Total survey completion, n (%)	Chi-square (*df*)	P value
**Non-Hispanic white (n=143)**						
	High school or less (N=28)	27 (96)	20 (71)	19 (68)	1.59 (2)	.12
	College graduate (N=74)	71 (96)	68 (92)	66 (89)	12.65 (2)	<.001
	Graduate school graduate (N=39)	39 (100)	38 (97)	38 (97)	13.34 (2)	<.001
**Hispanic (n=24)**						
	High school or less (N=5)	5 (100)	4 (80)	3 (60)	3.97 (2)	.21
	College graduate (N=14)	14 (100)	13 (93)	13 (93)	0.88 (2)	.17
	Graduate school graduate (N=5)	4 (80)	4 (80)	4 (80)	2.91 (2)	.07
**Black (n=59)**						
	High school or less (N=13)	13 (100)	9 (69)	7 (54)	2.15 (2)	.20
	College graduate (N=37)	36 (97)	29 (78)	26 (70)	1.21 (2)	.06
	Graduate school graduate (N=9)	8 (89)	8 (89)	6 (67)	1.16 (2)	.04
**Asian (n=35)**						
	High school or less (N=1)	0	1 (100)	1 (100)	—^a^	—
	College graduate (N=18)	15 (83)	15 (83)	15 (83)	3.1 (2)	.12
	Graduate school graduate (N=16)	0	16 (100)	16 (100)	3.09 (2)	.13

^a^Unavailable chi-square.

Among indices of SES, we found that there was a significant difference by education levels with respect to the completion of a partial survey and the entire survey. Although there was no significant difference between college graduates and graduate school graduates, participants with a high school diploma or less were less likely to complete the partial survey or the entire survey (OR 0.08, 95% CI 0.02-0.39, *P*=.001; OR 0.15, 95% CI 0.50-1.48, *P*<.001, respectively). There was no significant relationship between annual family income and the rate of proportionate completion.

We then explored survey participation by education level within each racial or ethnic group (Table 3). Among NHW participants, we found a significant difference by education level, that is, higher education was associated with more completion of a partial survey as well as the entire survey (χ^2^_2_=12.7, *P*<.001; χ^2^_2_=13.3, *P*<.001, respectively). Among black participants, there was a significant difference by education levels in total survey completion (χ^2^_2_=1.2, *P*=.04). There were no significant differences by education level in the Hispanic or Asian groups.

## Discussion

### Summary of Findings

We examined the rate of participation in a Web-based survey using convenience sampling strategies by different SES and racial or ethnic groups. We found that there were significant differences in the progress of Web-based survey participation among different groups in terms of race or ethnicity and education level. This is an important finding as the issue of health disparities is a major challenge in our health care system. It has been suggested that survey research results based on disproportionate participation by different portions of the population limit the applicability or generalizability of those results to the general population. Our results confirm reports from the literature that disproportional study attrition levels by different groups of race or ethnicity and education still exist for a Web-based survey.

We identified some trends in study participation of black participants. Of our different racial or ethnic groups, black participants were the least likely to consent compared with NHWs. Once they consented, they initiated the survey (there was no significant difference for initiation and partial survey completion); yet they were less likely to complete the entire survey. Furthermore, with publicly available study recruitment materials (flyer and online ads), black participants were likely to access the link to the screening questions, but they were less likely to consent to participate. However, once other minorities including Hispanic and Asian populations completed the screening questions and consented, they were likely to complete the survey. Even if internet accessibility has been increased across populations, our findings indicate a disproportionate distribution in the response rate.

### Comparison With Prior Work

There has been discussion of the historical barriers to participating in health research among black communities. Mistrust of health research is rooted in the mistreatment of black people by medical researchers [29], and this built mistrust still hinders the participation of blacks in health research [30]. In our study population, black participants were less likely to consent even if they completed the screening survey (which indicated that they were able to be reached by internet), and they were less likely to complete the entire survey. Although a Web-based survey has the potential to decrease health disparities in health research through its reach to a different proportion of the population, black populations might still be less likely to complete a survey even after they initiate it. Thus, we need to consider several factors (in addition to internet access) that influence the rate of study participation among black populations, including more limited access to resources, lower literacy levels, and a fear of disclosure [31]. These factors might continue to influence their perception of researcher disrespect and thus their decision as to whether to participate in Web-based survey research.

Biased findings based on limited representative sampling might lead to biased health recommendations, further deepening health disparities. Prior research has been focused on how to improve recruitment of underrepresented populations by addressing facilitators (eg, benefits to participants and cultural congruence) and barriers (eg, mistrust, stigma, and competing demands) [32]. Some researchers have posited that the use of a Web-based survey might diminish the issue of the disproportionate participants from different population [33]. Current research initiatives have been focused on improving health equity by involving underrepresented and disadvantaged members of the population in health research. Historically, racial or ethnic minorities are less likely to participate in any type of health research, and the health equity issue originates from unequal participation in health research, which forms the basis of health care [34]. Thus, research data are usually generated from the racial or ethnic majority, and the research findings are generally applied across racial or ethnic groups. The discrepancy in racial or ethnic different participation in health research has been a critical issue for the current health care system in the United States.

Moreover, health literacy is a significant issue in recruiting community residents to participate in health research. The different attrition rate we found based on the education indicator of SES is not surprising. In our findings, the level of education was related to the degree of survey completion, whereas annual family income was not. Moreover, this result is not just an issue of access to the internet because our study participants had internet access, but the attrition rate differed by the level of education. An interesting finding was the significant differences in survey completion by the level of education within the NHW and black subpopulations. Within the same racial or ethnic groups, disadvantaged individuals’ circumstances might hinder them from completing a Web-based survey. Health literacy in those with less education across race or ethnicity population might be overlooked if we focus only on racial or ethnic disparities. These differences in Web-based survey participation might be related to health literacy. Thus, we need to consider that disproportional survey participation is not only an issue of race or ethnicity. The difference by education level that we found is consistent with previous reports, which stated that individuals with lower education level were less likely to complete a Web-based survey [35]. We suggest that it would be related to electronic health (eHealth) literacy, which is defined as an ability to work with technology such as thinking of media-related issues, searching for numerous information, and making decisions based on the information [36]. Individuals with lower education levels might possess lower levels of both health literacy and eHealth literacy [37]. As health research methodology has been improved using more advanced technologies, we might need to consider eHealth literacy as a principal factor in the successful transition to the technology-based health research and health care outcomes.

### Implications

For further enhancement of participation by underrepresented populations in Web-based survey research, researchers need to consider enrichment as a strategy to build a relationship with the subpopulation of interest. On the basis of an awareness of the disproportionate distribution of the educational and racial or ethnic composition of study populations, researchers must develop strategies to improve their relationship with their participants. Technological barriers have been discussed in technology-based research [38], but such research has been focused on how to reach those subpopulations to improve their enrollment. We noted the different attrition levels of the subsample of our study participants. Survey researchers need to consider the racial or ethnic and educational level differences within their target population and the impact of these factors on Web-based survey participation.

Moreover, we cannot assume that it is easy for anyone from different demographic groups to complete a Web-based survey simply because they have a computer or a mobile phone and access to the internet. We need to promote the motivation to join a survey and support their completion of the survey. Web-based surveys might possess some advantages such as ease of distribution of the survey, utilization of images, and improvement of confidentially or anonymity. A clear description of the survey (including the study goals and example questions) and some features to encourage the completion of the survey such as images for low literacy groups, friendly reminders, secure access to the survey, and a progress bar might enhance survey completion. Community-based participatory research is considered a principal method for increasing the trust of and partnership with the community [39]. Building trust with communities is a strategy often used to encourage potential participants to engage in Web-based survey research [40]. Researchers might need to work with stakeholders in target communities to understand their needs and goals and to incorporate those items into the research [41]. As one of the main purposes of any research study is to be able to generalize the study results to the overall population through the use of a representative sample, we need to consider targeting underrepresented populations to encourage and promote their participation in a Web-based survey. Finally, we continuously need to make effort to improve eHealth literacy through understanding the users’ interaction with health technologies [42]. Thus, we can help the study participants complete the research tasks, use the health information, and therefore participate in making an impact on health care.

### Limitations

There are several limitations. As this was a secondary data analysis, we did not obtain demographic information (including gender, age, and SES) for those potential participants who only completed screening questions. These demographic factors could influence their motivation and decision to consent to participate and to complete the survey. We did not consider our survey content as a factor influencing the completion rate of the survey; however, some stress and depression measures, which were presented in the early portion of the survey, might have influenced the rate of continuation and completion of the survey. Moreover, most participants were female with young children (84.4%, 221/259), which is a group more likely to participate in a Web-based survey, thus our findings might not apply to males. We could not evaluate literacy level or other community-level characteristics to determine how those characteristics might influence the motivation for survey completion.

### Conclusions

Researchers need to understand that there is a significant difference between racial or ethnic groups as well as educational levels in terms of progress in Web-based survey participation. Public health research, especially community-based research, heavily relies on self-report and self-selection based on voluntary participation. Future researchers will need to make the effort to target underrepresented racial or ethnic groups and less educated populations to encourage their participation in Web-based survey research.

## Abbreviations

eHealth: electronic health
NHW: non-Hispanic white
OR: odds ratio
REDCap: Research Electronic Data Capture
SES: socioeconomic status
